# Towards Efficient Risky Driving Detection: A Benchmark and a Semi-Supervised Model

**DOI:** 10.3390/s24051386

**Published:** 2024-02-21

**Authors:** Qimin Cheng, Huanying Li, Yunfei Yang, Jiajun Ling, Xiao Huang

**Affiliations:** 1 School of Electronic Information and Communications, Huazhong University of Science and Technology, Wuhan 430074, China; m202172459@hust.edu.cn (H.L.);; 2 Department of Computer Science and Technology, Tsinghua University, Beijing 100084, China; 3 Department of Environmental Sciences, Emory University, Atlanta, GA 30322, USA

**Keywords:** risky driving detection, urban traffic safety, semi-supervised learning, AI and deep learning, intelligent transportation system

## Abstract

Risky driving is a major factor in traffic incidents, necessitating constant monitoring and prevention through Intelligent Transportation Systems (ITS). Despite recent progress, a lack of suitable data for detecting risky driving in traffic surveillance settings remains a significant challenge. To address this issue, Bayonet-Drivers, a pioneering benchmark for risky driving detection, is proposed. The unique challenge posed by Bayonet-Drivers arises from the nature of the original data obtained from intelligent monitoring and recording systems, rather than in-vehicle cameras. Bayonet-Drivers encompasses a broad spectrum of challenging scenarios, thereby enhancing the resilience and generalizability of algorithms for detecting risky driving. Further, to address the scarcity of labeled data without compromising detection accuracy, a novel semi-supervised network architecture, named DGMB-Net, is proposed. Within DGMB-Net, an enhanced semi-supervised method founded on a teacher–student model is introduced, aiming at bypassing the time-consuming and labor-intensive tasks associated with data labeling. Additionally, DGMB-Net has engineered an Adaptive Perceptual Learning (APL) Module and a Hierarchical Feature Pyramid Network (HFPN) to amplify spatial perception capabilities and amalgamate features at varying scales and levels, thus boosting detection precision. Extensive experiments on widely utilized datasets, including the State Farm dataset and Bayonet-Drivers, demonstrated the remarkable performance of the proposed DGMB-Net.

## 1. Introduction

The transportation industry has experienced significant advancements, resulting in a substantial increase in vehicle proliferation. While these advancements have certainly made life more convenient, they have also resulted in more crashes. As per the latest report from the World Health Organization (WHO) [1], crashes yield an estimated 20 to 50 million minor to moderate injuries and approximately 1.3 million fatalities each year. Additionally, several countries dedicate around 3% of their Gross Domestic Product to addressing the economic impact of crashes. Risky driving behaviors, including but not limited to cellphone use while driving, non-compliance with seat belt usage, speeding, drunk driving, distracted driving, and drowsy driving, have been identified as primary catalysts for these traffic incidents. The effective identification and penalization of such risky driving practices can contribute significantly to a reduction in the occurrence of crashes.

Risky driving behavior detection in traffic surveillance scenarios refers to the capture of drivers by electronic police devices on the roadway, followed by identifying dangerous and violating behaviors in the captured images. The significance of automatically detecting risky driving has garnered attention from several researchers in computer vision and artificial intelligence. Most existing studies utilize in-vehicle or dashboard cameras, capturing participants while they engage in specific distracted behaviors. These studies can be divided into image classification-based methods [2,3,4,5,6] and object detection-based methods [7,8]. Image classification-based techniques map input images into predefined categories of risky driving behavior [2,3,4,5,6]. In contrast, object detection-based techniques offer locational information about risky driving behavior through bounding boxes associated with specific body parts within the images [7,8]. Compared with the simulation scenario in the car, detecting risky driving in traffic surveillance scenarios is more challenging. This is due to varying data acquisition conditions in traffic surveillance scenes, such as complex illumination, extreme weather conditions, and diverse camera positions and angles. At present, various methods [9,10,11,12,13,14,15,16,17,18] have been developed to address specific challenges such as occlusion [9,10,11], adverse weather [15,16,17,18], or insufficient illumination [12,13,14] in traffic scenes. However, most of these methods are used for vehicle detection or autonomous driving, and only a few studies [12,13] have attempted to identify driver behavior. Moreover, both classification-based and detection-based methodologies heavily rely on abundant labeled data, with only a small fraction of research focusing on unsupervised learning for risky driving behavior classification tasks [19,20]. Notably, there is a lack of publicly reported semi-supervised or unsupervised learning approaches for object detection-based identification of risky driving behavior. Hence, it becomes imperative to explore and implement semi-supervised or unsupervised learning techniques in the domain of risky driving behavior detection.

Benchmark datasets serve a crucial role in advancing empirical progress within the realm of deep learning. Noteworthy benchmarks for risky driving detection include the State Farm dataset [21], AUC Distracted Driver dataset [22], FDUDrivers [23], and Drive&Act [24]. These existing datasets typically capture data related to the driver’s face, head, hands, or postures using in-vehicle cameras located at the front, back, side, or top of the vehicle. However, they significantly differ from data in traffic surveillance scenes in two key ways. Firstly, most of this data is simulated, requiring participants to perform specific actions. Secondly, images captured inside the vehicle generally have higher quality due to the closer shooting distance, fixed angles, and minimal impact from adverse weather and lighting conditions. Consequently, models trained on in-vehicle data may face challenges in effectively generalizing to road surveillance scenarios. Nonetheless, the development of such benchmarks remains limited, primarily due to considerations related to acquisition costs and privacy.

To rectify the scarcity of data associated with risky driving behaviors in traffic surveillance scenarios, this study proposes a novel benchmark known as Bayonet-Drivers. The foundational data for constructing Bayonet-Drivers is procured through an intelligent monitoring and recording system installed at road intersections. Data collection for Bayonet-Drivers covers various challenging conditions, including complex illumination, severe weather, and potential interference from car background information. Furthermore, due to the high prevalence of severe crashes attributed to disregarding seat belts and using phones while driving, the primary focus lies in detecting these specific types of risky driving behaviors. To our knowledge, this benchmark stands as the inaugural publicly available standard for the detection of risky driving behaviors within traffic surveillance settings. Therefore, Bayonet-Drivers can serve as a valuable tool for evaluating approaches geared towards the detection of risky driving behaviors within traffic surveillance contexts.

Additionally, to counter the issues resulting from an insufficient quantity of labeled data, DGMB-Net, a novel network architecture for the semi-supervised detection of risky driving behaviors, is proposed. The primary advantages of DGMB-Net can be broadly summarized into three aspects: (1) DGMB-Net incorporates an enhanced end-to-end teacher–student semi-supervised learning method, thereby reducing the burden associated with laborious and time-consuming data labeling. (2) The inclusion of an Adaptive Perceptual Learning(APL) Module enhances spatial perception and feature expression capabilities. This strategic integration ensures adequate capture of both local and global contexts within the network. (3) A Hierarchical Feature Pyramid Network(HFPN) is implemented, effectively amalgamating low-level and high-level features to generate comprehensive feature maps, thereby bolstering detection accuracy.

In conclusion, in order to address the limitations mentioned above, this study aims to provide the industry with a novel driver behavior benchmark and a high-precision, low-cost risky-driving-behavior detection method suitable for traffic monitoring scenarios. Due to the limited availability of traffic monitoring data in existing research, the adoption of fully supervised learning requires a large amount of cost. Hence, one of the innovations of this study is to provide a novel benchmark. This provides developers with data for monitoring scenarios, covering different challenging scenarios such as complex lighting, bad weather, etc., which helps them to conduct more in-depth research. The second innovation of this study is to provide a high-precision semi-supervised approach for risky-driving-behavior detection in traffic-monitoring scenarios. This approach not only reduces the cost of manual annotation during model training but also effectively addresses various challenging scenarios.

The structure of the remaining sections of this paper is organized as follows. Section 2 presents related work. Section 3 details the Bayonet-Drivers. Section 4 introduces the proposed semi-supervised DGMB-Net for driver risky-driving detection. Section 5 presents the analytical results, followed by the discussion in Section 6. Section 7 presents the conclusions and prospects.

## 2. Related Work

This section provides a concise summary of current risky driving behavior datasets and a discussion of representative vision-based risky driving behavior detection approaches in this field.

### 2.1. Datasets

Datasets serve as an indispensable resource for deep learning applications in the domain of computer vision. For the detection of risky driving behavior, this study categorizes publicly accessible datasets into four classifications based on the primary detection focus: the driver’s face, head, hands, and postures. Notably, the driver posture datasets, providing additional body cues, can be further segmented into various subcategories based on viewpoint and modality. Table 1 presents a comprehensive summary of these representative datasets for risky driving behavior detection, highlighting different aspects such as viewpoints, number of cameras used, focus, publication year, dataset scale, and image size. Below, an in-depth overview of several notable datasets is provided.

(1) State Farm Distracted Driver Dataset [21]: In 2016, the State Farm insurance company initiated a competition on Kaggle to detect distracted driver behavior, offering 102,150 images with a resolution of 640 × 480. The data were collected from a single viewpoint and modality, with the camera positioned to the side of the vehicle’s cockpit.
(2) AUC Distracted Driver Dataset [22]: This dataset was compiled using the rear camera of an ASUS ZenPhone (Model Z00UD), from which 17,308 frames were extracted and classified into ten categories. Similar to the State Farm dataset, the AUC dataset also employs a single viewpoint and modality.
(3) Driver Anomaly Identification Dataset (DAD) [25]: The DAD dataset consists of 783 min of video data, providing a multi-modal resource, alongside depth and infrared modalities, all with a resolution of 224 × 171. Furthermore, the DAD dataset offers multiple perspectives, including frontal and top views.
(4) Drive&Act [24]: Drive&Act is a comprehensive multi-view, multi-modal dataset that includes approximately 9.6 million frames. It captures infrared, color, 3D body pose data, and depth from six different views. Videos are meticulously labeled using a hierarchical annotation scheme, resulting in a total of 83 categories.

It is important to note that the majority of publicly available datasets focused on risky driving behaviors are gathered from in-vehicle cameras. However, there remains a significant gap in the availability of datasets related to risky driving behaviors observed within traffic surveillance scenarios.

**Table 1:** Publicly accessible datasets for driver distracted detection.

Dataset	Camera Viewpoints	Num of Cameras	Target	Year	Size	Resolution
DrivFace [26]	front	1	Driver face	2016	606 images	640 × 480
VIVA-Face [27]	front	1	Driver face	2016	39 video clips	544 (height)
Pandora [28]	simulated	1	Driver head	2017	110 video clips	640 × 480
DriveAHead [29]	front	1	Driver head	2017	21 video clips	512 × 424
DD-Pose [30]	front, back	2	Driver head	2019	660 k images	—
VIVA-Hands [31]	front, back, side, top	1	Driver hands	2015	11 k images	—
Turms [32]	front, bottom	1	Driver hands	2018	14 k frames	640 × 240
State Farm [21]	side	1	Driver postures	2016	22,424 images	640 × 480
AUC [22]	side	1	Driver postures	2019	14,478 frames	1080 × 1920
EEE BUET [33]	front	1	Driver postures	2018	2 × 312 video clips	854 × 480
DAD [25]	top, front	3	Driver postures	2021	783 min videos	224 × 171
FDU Drivers [23]	front	1	Driver postures	2020	20,000 images	224 × 224
Drive&Act [24]	top, front, back	6	top, front, back	2019	9.6 M frames	—

### 2.2. Sensor-Modal Data-Based and Multimodal Data-Based Method

The performance of a vehicle is directly influenced by the driver’s behavior, and this impact can be assessed through the analysis of single-modal data from vehicle motion sensors. Espino-Salinas et al. [34] addressed the identification of drivers through motor activity generated by the main elements of the vehicle through genetic algorithms. With the advancement of sustainable multi-sensor collection techniques, numerous studies have been undertaken to integrate and fuse data from multiple sensors. Du et al. [35] verified that an improved predictive performance for distraction detection could be achieved by integrating facial expression, speech, and vehicle signals. Streiffer et al. [36] devised a comprehensive data collection and analysis framework called DarNet. This system utilized convolutional neural networks (CNNs) for analyzing driving image data and recurrent neural networks for processing inertial measurement units sensor data. Ultimately, the integration of the two outputs was accomplished through Bayesian networks. Rashwan et al. [37] introduced a two-stage model, which firstly involved three independent modules for feature extraction from audio, image, video, and other signals. Subsequently, an estimation of the driver’s risky state, based on the hidden Markov model, was generated. Ultimately, the outputs and contextual information from each module were fused using a Bayesian network. Zhang et al. [38] proposed a deep unsupervised multi-modal fusion network composed of three main modules: multi-modal representation learning, multi-scale feature fusion, and unsupervised driver-distraction detection for driver-distracted detection. Gao et al. [39] introduced the M2-Conformer, a hybrid framework integrating Transformer and CNN architectures in parallel branches, for extracting driving scene and vehicle dynamics features. Co-occurrence features are subsequently input into a customized Feature Aggregation Module to generate higher-quality aggregated features.

### 2.3. Vision-Based Risky Driving Detection

Early methodologies [40,41] commonly utilized artificial feature extraction techniques such as Local Binary Pattern (LBP) and Histogram of Oriented Gradients (HOG) for the identification of risky driving behaviors. These extracted features were subsequently processed using classifiers like Support Vector Machines (SVMs) for classification tasks. However, in recent decades, there has been a significant shift in focus towards deep learning-based approaches, attributed largely to their superior feature representation capabilities. The field of vision-based recognition of risky driving behaviors can be broadly bifurcated into two principal methodologies: image classification-based [2,3,4,5,6] and object detection-based [7,8].

Image classification-based method: Image classification-based methodologies strive to classify input images into predefined categories that correspond to risky driving behaviors. Yan et al. [42] focused on locating the driver’s hand by extracting prominent information, with the goal of predicting driving posture via trainable filters and local neighborhood pooling operations. Meanwhile, Li et al. [43] designed a lightweight network, termed OLCMNet, to detect driver distractions. They accomplished this by extending feature maps into two separate branches via point-wise convolution, effectively reducing network size and enhancing real-time performance. In a separate work, Abouelnaga et al. [44] integrated ensemble learning, specifically a genetic algorithm, to improve the accuracy and generalization ability of detection methods. They performed a precisely weighted summation of outputs from a diverse ensemble of networks, with each network in the ensemble trained on different input modalities, such as raw images, hand images, face images, and the fusion of face and hand images. Subsequently, Eraqi et al. [22] extended the dataset and simultaneously augmented the capacity of the neural network to enhance the generalization and robustness of their algorithms across diverse scenarios.

Object detection-based method: Methods utilizing object detection strive to pinpoint and accurately identify instances of hazardous driving behaviors within designated input images. Numerous researchers have concentrated their efforts on enhancing the robustness of detection networks. As an illustration, Sajid et al. [8] proposed an innovative detection framework that incorporates a weighted bidirectional feature fusion network and a hybrid augmentation technique. This approach identifies objects associated with risky driving activities and determines the regions of interest corresponding to specific body parts. Certain research endeavors have specifically targeted the detection of particular behaviors, such as seat belt violation [45,46,47] and cell phone use [48,49]. These behaviors are given emphasis due to their strong correlation with severe crashes. Hoang et al. [48], for instance, detected mobile phone usage by identifying the position of the driver’s hands on the steering wheel and determining any hands-off-wheel instances. In identifying seat belt violations, most research studies have adopted a two-step process: initial segmentation of the windshield region, followed by detection of seat belt presence. Elihos et al. [50] utilized single-shot multi-box object detection techniques to identify the windshield and passenger area and then proceeded to verify the presence of seat belt violations. However, the researchers acknowledged the restricted real-time performance of their methodology. To address this limitation, Yang et al. [45] executed pruning and quantization of SSD MobileNet V2 to detect the driver’s seat belt. Similarly, Chun et al. [46] utilized a feature pyramid network (FPN) with multiple detection heads to estimate body posture and identify seat belts. Additionally, Feng et al. [47] exploited the spatial relationship of the front windshield to locate it, before applying the Hough transform to establish the windshield boundary. This led to a successful differentiation of the positions of the driver and passenger, thus facilitating seat belt recognition. Despite these substantial strides in seat-belt-violation detection, it is important to note that these studies have been conducted on external vehicle data, and these datasets have not been made publicly available to date.

Current methodologies, whether predicated on image classification or object detection, significantly depend on voluminous amounts of labeled data. This requirement often leads to substantial labor costs. Aiming to circumvent this limitation, several researchers have directed their efforts towards unsupervised recognition of risky driving behaviors [19,20]. Li et al. [19], for instance, introduced an unsupervised deep learning algorithm, referred to as UDL. This algorithm is designed for fine-grained classification of driver distraction behaviors. Concurrently, Roy [20] developed an unsupervised low-rank non-negative dictionary and applied a threshold-based reconstruction error criterion. This approach enables the detection of drivers using mobile phones, based on their proposed driving dataset. Both studies focus on classification-based detection of risky driving behaviors. Despite these developments, it is important to note that as of now, there is no publicly available research that addresses semi-supervised or unsupervised learning for object detection-based recognition of risky driving behaviors.

## 3. Bayonet-Drivers Dataset

The entirety of the original data for Bayonet-Drivers was procured utilizing high-definition intelligent integrated cameras, integrated within an intelligent monitoring and recording system situated at a road intersection. The cameras are positioned along a main road with three or four lanes, situated at a height of approximately 4.5 m above typical vehicles and roughly 6 m above ground level.

**Figure 1:**
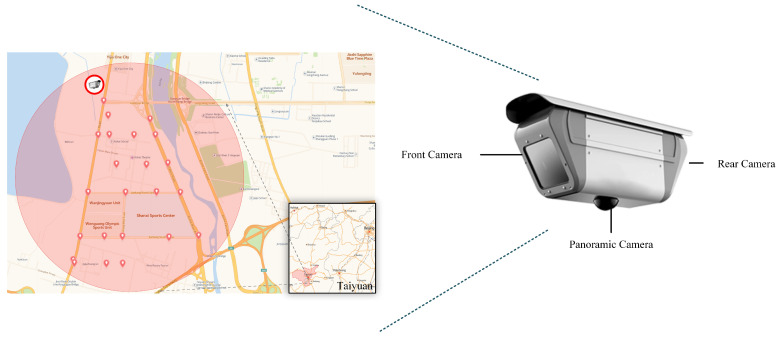
The details of high-definition intelligent integrated camera and study region.

The site of data collection is situated within the Jinyuan District of Taiyuan City, Shanxi Province, China, an area that spans 289 square kilometers and encompasses a total of 537 km of roads (as depicted in Figure 1). This endeavor resulted in a compilation of 100 h of video clips. To ensure a diverse dataset, video capture was executed at various times, specifically between 9:00 and 15:00 and from 19:00 to 20:30 during July 2020. Due to the long time, wide geographical range, and strong randomness of the data collection, Bayonet-Drivers encompasses individuals of varying ages (including the young, middle-aged, and some elderly) with diverse driving habits. According to the most recent report from the WHO as of December 2023 [1], drivers who use mobile phones are about four times more likely to be involved in a crash than those who do not. Using a cell phone while driving slows down reaction times (especially braking reaction time, but also reaction time to traffic signals) and makes it difficult to stay in the right lane and maintain the right following distance. Wearing seat belts reduces the risk of death for vehicle passengers by 50%. Consequently, Bayonet-Drivers comprises scenarios of safe driving and risky driving, where risky driving includes using a cell phone and not wearing a seat belt. Details of Bayonet-Drivers are shown in Table 2.

As the data were gathered in a real-world setting, Bayonet-Drivers encapsulates a broad spectrum of challenging scenarios, including a variety of weather conditions such as sunny, cloudy, and foggy days, as well as complex illumination conditions like low light, dazzling light, and uneven illumination, along with interference from the car interior. Moreover, different forms of partial occlusions obscure the driver’s posture. For instance, sun visors may entirely or partially obstruct the driver’s face, hindering the detection of cell phone usage.

**Figure 2:**
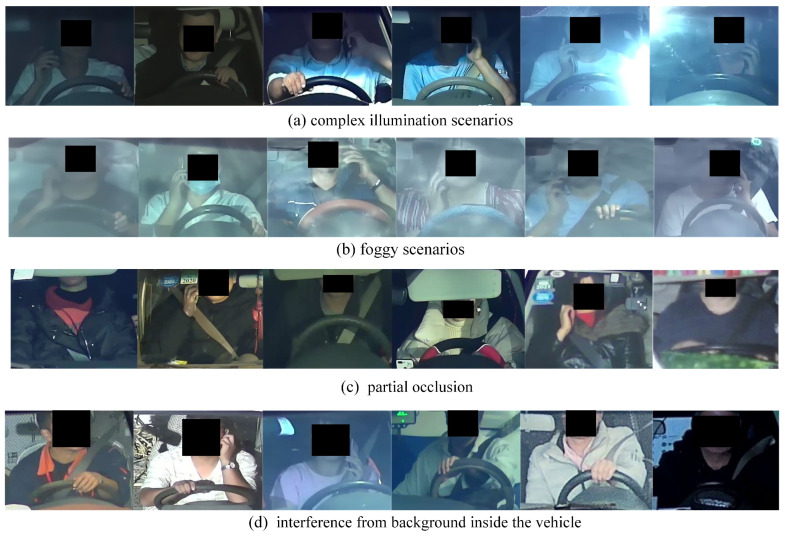
Some challenging example images in Bayonet-Drivers.

**Figure 3:**
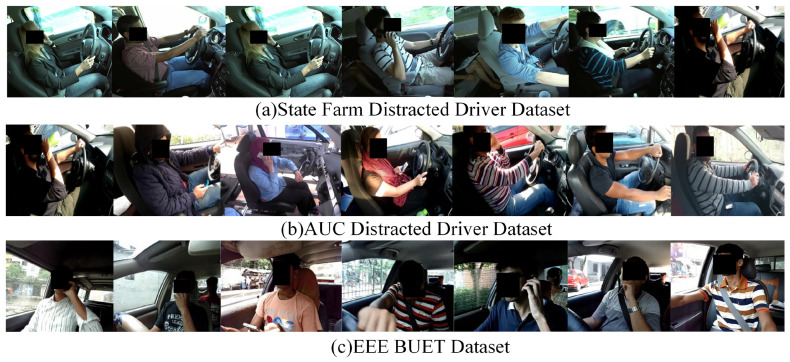
Some typical examples of three popular in-vehicle datasets.

The images within the Bayonet-Drivers dataset possess dimensions of 224 × 224 pixels. During the construction process, an image was extracted every 30 frames for regular scenarios, while for challenging scenarios, an image was extracted every 10 frames. This resulted in a final dataset comprising 10,000 images, with 3000 of them annotated in the MS COCO format. Figure 2 displays some of the challenging example images from the Bayonet-Driver dataset. For comparison, examples from several representative publicly available in-vehicle datasets, including State Farm [21], AUC [22], and EEE BUET [33], are illustrated in Figure 3.

## 4. Methodology

**Figure 4:**
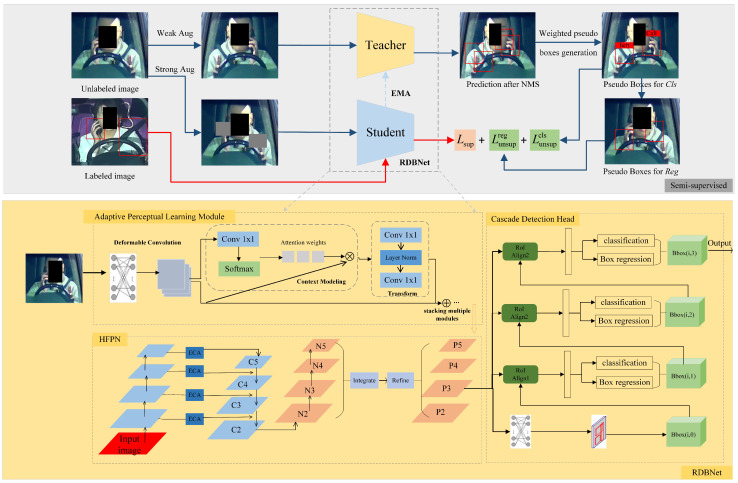
Overall architecture of DGMB-Net.

As shown in Figure 4, an end-to-end semi-supervised network for risky driving detection termed by DGMB-Net based on the classical teacher–student framework [51] is proposed. Within DGMB-Net, the teacher and student models employ the same structure, specifically RDB-Net, which is composed of the Adaptive Perceptual Learning (APL) module, the Hierarchical Feature Pyramid Network (HFPN), and the cascade detection head. While the APL module and HFPN are designed to improve detection accuracy through advancing spatial perception and fusing features at different levels and scales, the cascade detection head is introduced to realize high-precision bounding box regression and object classification.

### 4.1. Semi-Supervised Learning

The teacher model and student model have the same structure as mentioned previously. The teacher model produces pseudo-labels for unlabeled images, while the student model is simultaneously trained on labeled images with ground-truth labels and unlabeled images.

Both the teacher model and the student model are randomly initialized throughout the training phase. During each training iteration, a training data batch is formed by randomly sampling labeled and unlabeled images based on a certain data sampling ratio. Unlabeled data are processed by weak augmentation and strong augmentation, aiming to increase the diversity and variation of the unlabeled data, thereby improving the performance and generalization of the model. Weak augmentation, such as random cropping and color jittering, is applied for pseudo-labeling of the teacher model and training of the student model. Strong augmentation such as rotation, scaling, shearing, and flipping is utilized for the detection training of student models. Throughout the training phase, the student model is trained using gradient descent, while the teacher model is continually updated based on the student model using the commonly employed exponential moving average strategy. Afterwards, Non-Maximum Suppression (NMS) is usually utilized to remove the large number of pseudo boxes that are generated by the teacher model and have lower confidence than a fixed threshold.

Although the process of NMS can eliminate the majority of non-foreground boxes, there may still be some redundant boxes remaining due to the overlap between the actual targets and the generated pseudo-labels in terms of their spatial location, dimensions, or visual characteristics. In this case, simply applying one threshold to filter out these redundant candidate boxes might result in inaccurate boundary delineation or even missing detection. To address this problem, a Nonlinear Weighted Pseudo Boxes Generation (NWPG) algorithm is proposed to align the generated pseudo boxes with the ground truth for the accuracy of pseudo-labels. The following is the generation process:

(1) $$X=\frac{\sum_{i=1}^{n}x_{i}\cdot sqrt\left(w_{i}\right)}{\sum_{i=1}^{n}sqrt(w_{i})}$$

(2) $$Y=\frac{\sum_{i=1}^{n}y_{i}\cdot sqrt\left(w_{i}\right)}{\sum_{i=1}^{n}sqrt(w_{i})}$$

where *X*, *Y* are the final coordinate values, respectively. NWPG only calculates the coordinates of the upper left and the lower right corners of each candidate box. $x_{i}$ and $y_{i}$ respectively represent the initial coordinate value, and $w_{i}$ represents the confidence score of the corresponding candidate box.

The loss function *L* is the weighted sum of the supervised loss function $L_{\sup}$ and the unsupervised loss function $L_{un\sup}$:

(3) $$L=L_{\sup}+\alpha L_{un\sup}$$

where α controls the proportion of unlabeled image loss, and both $L_{\sup}$ and $L_{un\sup}$ are normalized by the number of images in their respective training data:

(4) $$L_{\sup}=\frac{1}{N_{s}}\sum_{i=1}^{N_{s}}\left(L_{cls}\left(I_{s}^{i}\right)+L_{reg}\left(I_{s}^{i}\right)\right)$$

(5) $$L_{un\sup}=\frac{1}{N_{u}}\sum_{i=1}^{N_{u}}\left(L_{cls}\left(I_{u}^{i}\right)+L_{reg}\left(I_{u}^{i}\right)\right)$$

where $L_{cls}$ represents the classification loss, $L_{reg}$ represents the bounding box regression loss, $I_{s}^{i}$ represents the i-th labeled image, $I_{u}^{i}$ represents the i-th unlabeled image, $N_{s}$ represents the total number of labeled images, and $N_{u}$ represents the total number of unlabeled images.

### 4.2. RDB-Net

RDB-Net is composed of three modules: the Adaptive Perceptual Learning Module for feature extraction, the Hierarchical Feature Pyramid Network for feature fusion, and the cascade detection head for high-precision detection.

#### 4.2.1. Adaptive Perceptual Learning Module

Risky-driving-behavior detection in traffic monitoring scenarios is a challenging task due to the presence of complex lighting conditions, adverse weather conditions, and interference from the background inside the vehicle. To enhance the adaptability of feature extraction network in different scenarios, a plug-and-play module, termed Adaptive Perceptual Learning (APL) Module, is proposed.

**Figure 5:**
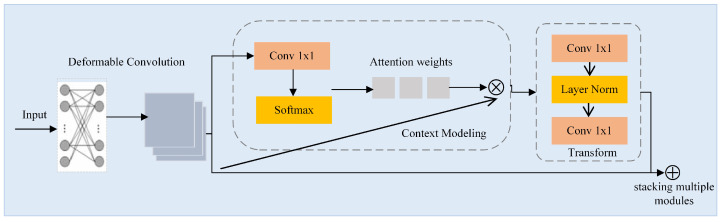
The structure of the APL module.

For the accuracy and versatility of the model, the classical ResNet50 is applied as the base backbone. The APL module is incorporated into the final three stages of the ResNet50 network. Within the APL module, deformable convolution (DCN) [52] effectively captures fine details and effectively models spatial variations in complex lighting and noisy conditions by adaptively adjusting the receptive field. Simultaneously, the APL module leverages global context (GC) modeling [53] to address the challenges of complex lighting and noisy images by integrating overall information and capturing remote dependencies, thus compensating for the loss of detail and low contrast. Figure 5 shows the structure of the APL module. Firstly, deformable convolution processes the feature map of the previous stage. Then, the feature map is passed through a 1×1 convolution block and softmax function in the context modeling part and is then added to the original input to acquire the global context feature, which expands the input receptive field. In the transformation process, two convolutional layers are employed to decrease the channel dimension and minimize the parameter count. Finally, the result and input of the feature transformation are added element-wise. The process can be expressed with the subsequent formula:

(6) yi=xi+W2ReLU(LN(W1∑j=1NpeWkxj∑m=1NpeWkxmxj))

where *x* is the feature map input, *y* is the output, *i* is the index of positions, *j* represents traversing all positions, $N_{p}$ is the total number of positions in the feature maps, $W_{k}$ represents the weight through the first 1 × 1 convolution, $W_{1}$ represents the weight through the first 1 × 1 convolution after the softmax function, $W_{2}$ represents the weight through the last 1 × 1 convolution, $\alpha_{j}=\frac{e^{w_{k}x_{j}}}{\sum_{m=1}^{N_{p}}e^{w_{k}x_{m}}}$ is the global attention pooling weight, and δ(·)=W2ReLu(LN(W1(·))) is the transformation process.

#### 4.2.2. Hierarchical Feature Pyramid Network

**Figure 6:**
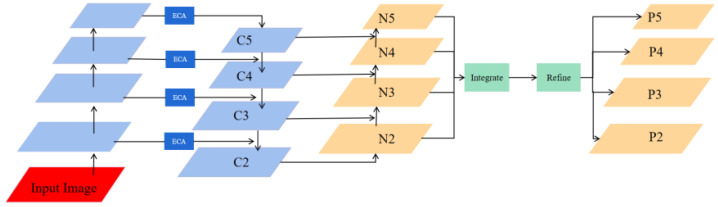
The structure of HFPN.

Regarding the FPN module, it strengthens the features extracted by the backbone, enabling the model to detect targets of different scales efficiently. However, traditional FPN’s sole reliance on a top-down path hampers the balanced distribution of feature information across levels, limiting the impact of low-level features on high-level features. To effectively capture multi-scale feature information and perform hierarchical feature fusion, this study proposes a Hierarchical Feature Pyramid Network (HFPN) inspired by [54,55]. Figure 6 illustrates the workflow of HFPN. Firstly, semantic information is propagated through a top-down pathway. Subsequently, location information is propagated through a bottom-up pathway to facilitate feature fusion. Additionally, an Efficient Channel Attention (ECA) block [56] is incorporated into the input part of HFPN to adjust the weight of features adaptively. Finally, a balanced integration of features from layers N2, N3, N4, and N5 is performed.

The following are the processes to balance the features at each level: The first step is to generate balanced semantic features. Assuming that the resolution of the features has four levels {N2,N3,N4,N5}, with N2 having the highest resolution. Then, {N2,N3,N4,N5} are adjusted to have the same size as N4 using interpolation, and the maximum pooling and balanced semantic features are achieved using the formula:

(7) $$C=\frac{1}{L}\sum_{l=l_{\min}}^{l_{\max}}N_{l}$$

where *L* is the number of feature levels, and $l_{\max}$ and $l_{\min}$ represent the index of the highest and lowest feature level, respectively. The refined feature is then used to enrich the feature details using non-local operation. To obtain both low-level and high-level features, the extracted features are rescaled using the same procedure but in reverse to output P2, P3, P4, and P5.

#### 4.2.3. Cascade Detection Head

**Figure 7:**
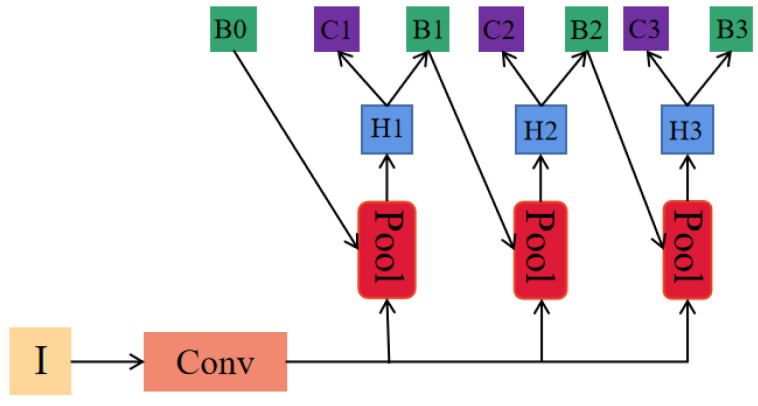
Structure of cascade detection head.

A cascade detection head was suggested to address the limitation of traditional networks in improving accuracy, arising from the use of a single threshold setting. The cascade detection head converts the traditional bounding box regression task into a cascaded regression task, and multiple detectors are cascaded after the Region Proposal Network (RPN). This work adopts the cascade detection head as the detection head of RDBNet. The structure is shown in Figure 7.

## 5. Experiment and Analysis

In this section, ablation experiments are executed to analyze the contributions of the APL module. Then, the proposed HFPN is compared with the baseline FPNs. Afterwards, this study verified the effectiveness of the proposed semi-supervised method by comparing it with the fully supervised method and other semi-supervised methods. Then, DGMB-Net is compared with several baseline networks. Finally, this study visualized the performance of DGMB-Net.

### 5.1. Dataset and Experiment Settings

All experimental evaluations were carried out using the State Farm dataset and the Bayonet-Drivers dataset. Given that the State Farm dataset is primarily tailored for classification tasks, this work carried out the annotation of images depicting risky driving behaviors. Specifically, these behaviors included right-handed and left-handed cellphone use, both in texting and phone use scenarios, annotated in the COCO format. Ultimately, the State Farm dataset used in this study contained 9256 images, with 2776 of them labeled. The Bayonet-Drivers dataset comprised 10,000 images, with 3000 labeled images.

The experimental setup included the use of an Nvidia Geforce 1080Ti 11GB graphics processing unit. The operating system and deep learning frameworks employed were Ubuntu18.04 and PyTorch 1.7.0, respectively. The parameters for the experiments were set as follows: the first 500 iterations adopted a linear learning rate strategy, where the initial learning rate was set at 0.001. Following this, the learning rate was adjusted to 0.01 and was subsequently reduced by a factor of 0.1 every 40,000 iterations. In the context of supervised learning, the batch size was fixed at 4. For semi-supervised learning, the batch size was increased to 5, maintaining a ratio of labeled to unlabeled images of 1:4. The total number of iterations performed was 180,000.

### 5.2. Metrics

This study used the COCO metrics [57] as evaluation metrics, which is a common evaluation standard for object detection. Among COCO metrics, this study employed mAP, mAP@0.5, mAP@0.75, $AP_{M}$, and $AP_{L}$ to evaluate the performance of the model, and their calculation formulas are detailed as follow:

(8) $$P=\frac{TP}{TP+FP}$$

(9) $$R=\frac{TP}{TP+FN}$$

(10) $$AP=\int_{0}^{1}P(r)dr$$

(11) $$mAP=\frac{1}{N}\sum_{i=1}^{N}AP_{i}$$

TP represents true positives, signifying actual positives correctly classified by the classifier. FP stands for false positives, denoting actual negatives incorrectly classified as positives. FN represents false negatives, indicating actual positives incorrectly classified as negatives. TN denotes true negatives, representing actual negatives correctly classified as negatives by the classifier. AP, calculated as the area enclosed by the curve when precision is plotted against recall, serves as a pivotal metric in object detection. A higher AP signifies superior performance. mAP represents the mean average precision, calculated as the average of the *AP* values for all classes. It serves as a common metric for measuring the overall performance of an algorithm. In the mAP calculation formula, $AP_{i}$ represents the AP value for the class with index *i*, and *N* denotes the number of classes. mAP@0.5 denotes the average precision when the Intersection over Union (IoU) is set to 0.5. mAP@0.75 denotes the average precision when the Intersection over Union (IoU) is set to 0.75. $AP_{M}$ and $AP_{L}$ are selected as the evaluation indices for medium and large targets, respectively.

### 5.3. Results and Analysis

#### 5.3.1. Ablation Experiments of Adaptive Perceptual Learning (APL) Module

We conducted ablation experiments on the proposed Adaptive Perceptual Learning (APL) Module. The ablation experiments adopt the semi-supervised learning method mentioned in Section 4.1. The results of ablation experiments on two datasets are shown in Table 3 and Table 4. The ablation experimental results show that the newly added GC (Global Context) module significantly improves the AP metric. This is attributed to the potent global modeling capabilities of the GC module, which optimizes feature representation. Additionally, the inclusion of deformable convolution contributes to the enhancement of the AP value. This is attributed to the deformable convolution’s ability to flexibly adjust the receptive field, allowing for better adaptation to changes in the target, such as seat belts. This study conducted experiments on different CNNs to select the most appropriate CNN. The experimental results show that the mAP of ResNet50 and ResNeXt101 are very close, while the network parameters of ResNeXt exceed ResNet50. So this work chose ResNet50 as the backbone.

#### 5.3.2. Effects of Hierarchical Feature Pyramid Network (HFPN)

This section compared the effect of HFPN and several FPN baselines with excellent performance, specifically, FPN [58], BiFPN [59], PAFPN [54], and BFP [55]. FPN is the most primitive architecture, BiFPN, PAFPN, and BFP are all developed on it. Among them, BiFPN is a weighted bidirectional feature pyramid network. Compared with ordinary FPN, PAFPN adds a bottom-up path to enhance the positioning ability on multiple scales. The main innovation of BFP is to use the same deeply integrated balanced semantic features to enhance multi-level features. The semi-supervised learning method mentioned in Section 4.1 is applied in this section.

Table 5 and Table 6 tabulate the experimental results on Bayonet-Drivers and State Farm datasets, respectively. It shows that HFPN outperforms BFP by 3.3 AP points on Bayonet-Drivers with only a 3.54 M parameter increase. Moreover, there is a notable improvement in both $AP_{L}$ and $AP_{M}$ values, with an increase of 0.9 and 2.5, respectively. This improvement is attributed to HFPN’s capacity to not only focus on crucial feature channels but also effectively integrate multi-scale features from different levels, thereby enhancing the model’s ability in object detection.

#### 5.3.3. Comparison of DGMB-Net with Other Semi-Supervised Methods

In this section, we undertook a comparison between DGMB-Net and other semi-supervised methodologies. RDB-Net is utilized as the detection model. Experiments were conducted using varying labeled ratios on both the Bayonet-Drivers and State Farm datasets. The ratios of 1%, 5%, 10%, 20%, and 30% represent the proportion of labeled images in relation to the total dataset. Table 7 presents the mAP values of both DGMB-Net and other semi-supervised learning methodologies.

As can be discerned from the experimental results in Table 7, all the semi-supervised methods showed a significant improvement over the supervised method. DGMB-Net outperforms the supervised method by 26.7 points, 25.2 points, and 11.8 points when there are 1%, 5%, and 10% labeled data, respectively. Moreover, it becomes evident that the proposed semi-supervised learning method has led to improvements in the mAP value when compared to other state-of-the-art methods. This can be attributed to the design of the Nonlinear Weighted Pseudo Boxes Generation algorithm, which aligns the generated pseudo boxes with the ground truth, thereby enhancing the accuracy of pseudo labels. Specifically, DGMB-Net outperforms the E2E by 3.5 points, 2.2 points, and 1.1 points when there are 1%, 5%, and 10% labeled data on Bayonet-Drivers, respectively. Notably, the semi-supervised learning methodology demonstrates a greater advantage when the label ratio is smaller. When the entire dataset is employed for training, DGMB-Net attains mAP values of 54.5 and 72.3 on the Bayonet-Drivers and State Farm datasets, respectively.

#### 5.3.4. Comparison with Mainstream Detectors

Several classic detection networks, single-stage Yolox [63] and Retinanet [64] and two-stage Fast R-CNN [65], Faster R-CNN [66], and Cascade R-CNN [67], were compared with DGMB-Net on Bayonet-Drivers and State Farm datasets. Table 8 and Table 9 report the experimental results. As for two-stage methods, the AP of RDB-Net on Bayonet-Drivers is 14.9 percentage points, 4.3 percentage points, and 2.7 percentage points higher than Fast R-CNN, Faster R-CNN, and Cascade R-CNN, respectively. As for single-stage methods, the AP of RDB-Net on Bayonet-Drivers is 9.8 percentage points, 1.0 percentage points higher than Yolox and Retinanet. Although early algorithms (Fast R-CNN and Faster R-CNN) had fewer parameters, their AP value can only achieve 36.3 and 46.9 on Bayonet-Drivers. However, the parameters of the RDB-Net only increased by 5.89 M compared to Cascade R-CNN. At the same time, after introducing semi-supervised learning, the performance of RDB-Net has been further improved. DGMB-Net boasts the highest detection precision and the most optimal comprehensive detection performance.

#### 5.3.5. Visualization Results of DGMB-Net

**Figure 8:**
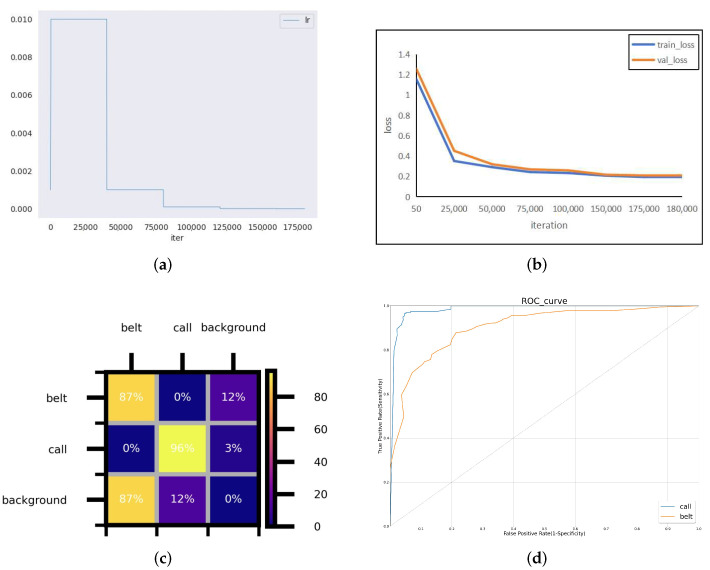
Performance diagrams for DGMB-Net using the Bayonet-Drivers dataset: (**a**) learning rate, (**b**) train and validation loss, (**c**) confusion metrics, (**d**) Receiver Operating Characteristic (ROC) curve.

**Figure 9:**
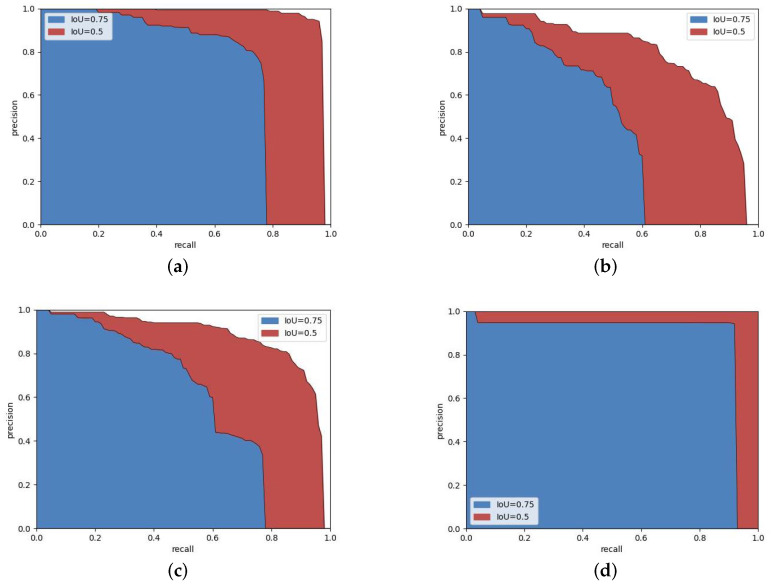
Precision–recall curve (PR Curve) for different datasets and different categories: (**a**) calls in Bayonet-Drivers dataset, (**b**) belt in Bayonet-Drivers dataset, (**c**) all classes in Bayonet-Drivers dataset, (**d**) all classes in State Farm dataset.

This section performed a visual analysis of DGMB-Net. Figure 8 shows performance diagrams for DGMB-Net on the Bayonet-Drivers dataset. The confusion matrix shows that some errors occur; for example, belt and call were misclassified into background categories, resulting in missed detection. The ROC curve for the DGMB-Net is given in Figure 8d. The ROC curve showed that DGMB-Net achieved good results in both belt and call categories. Figure 9 shows the precision–recall curves (PR Curve) for different datasets and different categories. As is demonstrated in Bayonet-Drivers, DGMB-Net performed better on the detection of phone call than that of belt. One possible cause is the deformation of belt during the driving. In addition, the background information such as the color of clothes can interfere with the detection.

**Figure 10:**
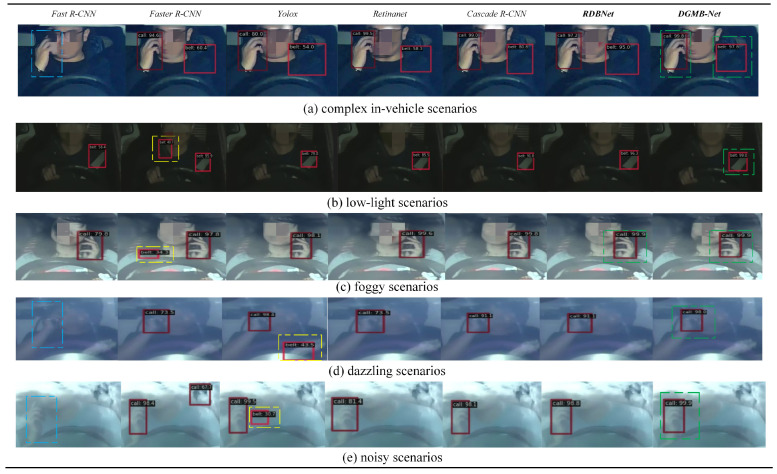
Some examples of detection results on the Bayonet-Drivers dataset.

**Figure 11:**
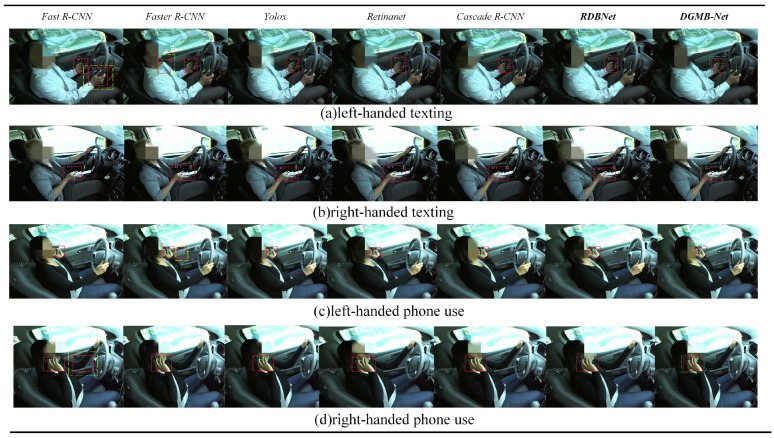
Some examples of detection results on the State Farm dataset.

Figure 10 and Figure 11 show examples of detection visualization results of images on Bayonet-Drivers and State Farm datasets, respectively. The dashed boxes in blue, yellow, and green represent missed detection, wrong detection, and correct detection with the highest confidence, respectively. It is shown that the proposed DGMB-Net successfully addresses the aforementioned challenges. As shown in Figure 10a, in complex in-vehicle background (the color of clothes and the color of seat belt are very similar), Fast R-CNN missed detection. Furthermore, Faster R-CNN, Yolox, and Retinanet exhibited low confidence, whereas DGMB-Net achieved the highest confidence. To verify the generalization capability of DGMB-Net, this study also conducted visualization experiments on the State Farm dataset. It is evident that DGMB-Net demonstrates outstanding detection results in the in-vehicle environment. Simultaneously, it effectively addresses the occlusion challenge posed by left-hand phone usage.

## 6. Discussion

### 6.1. Advantages and Limitations

Current vision-based methods [2,3,4,5,6] for detecting risky driving behaviors primarily focus on scenes within vehicles. They commonly employ a single or integrated CNN for direct identification of risky or distracted behaviors, aiming to alert drivers and improve safety. Different from them, this study can deal with different challenging scenes on the real road captured by surveillance cameras by using the APL module and HFPN. Moreover, the majority of methods proposed for detecting risky driving behaviors relies on supervised learning [7,8], requiring a substantial volume of labeled data for efficient training. However, obtaining such labeled data consistently poses a challenge in developing effective data collection strategies. Furthermore, manually labeling driving data is not only relatively expensive and time-intensive but is also subject to human judgment. In contrast, this study employs an end-to-end semi-supervised learning approach, reducing labeling costs while maintaining detection accuracy.

However, there are some limitations in this study. The risky behaviors identified in this study included not wearing a seat belt and answering a phone. The experimental results reveal high accuracy in detecting phone usage but a lower accuracy in identifying instances of not wearing a seat belt. This discrepancy reduces the overall detection accuracy. The challenge arises from seat belts undergoing deformation and being susceptible to interference from background elements, such as clothing color. To address this, the introduction of deformable convolution is proposed to handle seat-belt deformations. Additionally, addressing challenges posed by seat belt colors resembling clothing colors is essential for future improvements. At the same time, although the detection accuracy of the method proposed in this study is higher than that of other detection models [63,64,65,66,67], the number of parameters in the model is also slightly increased, which has a certain impact on real-time performance. In the future, how to further lightweight the network should be considered.

### 6.2. Implications

The findings of this study hold significant implications for the detection of risky driving behavior within the intelligent transportation industry. Firstly, this study introduces a novel dataset named Bayonet-Drivers, categorizing drivers into two groups: safe driving and unsafe driving. The latter encompasses behaviors with a high likelihood of causing crashes, specifically, failure to wear seat belts and phone calls. Bayonet-Drivers spans challenging scenarios, including complex illumination, severe weather, and potential interference from surrounding vehicle information. Applying Bayonet-Drivers in real-time traffic monitoring supports the enhancement of efficient driver-behavior-detection algorithms for future intelligent transportation systems, so as to further regulate the driver’s behavior and ultimately improve road safety.

Additionally, this study establishes a risky-driving-behavior detection approach, DGMB-Net. DGMB-Net greatly reduces manual labeling costs by semi-supervised learning. By combining the APL module and HPN module, DGMB-Net can effectively deal with different challenging scenarios in reality. Components like deformable convolution and global context blocks can be selectively applied based on specific needs. Implementing DGMB-Net in intelligent traffic monitoring enables automated detection of risky driving. Upon detecting risky behaviors, the monitoring system will promptly feedback the data to the traffic management department, initiating timely driver reminders and penalties.

## 7. Conclusions and Prospect

This study introduces Bayonet-Drivers, the pioneering benchmark for detecting risky driving behaviors in traffic surveillance contexts. This comprehensive framework comprises many challenging scenarios, providing an invaluable standard for both the evolution and appraisal of methodologies aimed at detecting risky driving behaviors. In addition, DGMB-Net—a novel semi-supervised network architecture—is proposed specifically for the detection of risky driving behaviors. The DGMB-Net incorporates an enhanced semi-supervised learning approach designed to navigate the costly challenge of data labeling. It also integrates an Adaptive Perceptual Learning Module and a Hierarchical Feature Pyramid Network to preserve detection accuracy. This study undertook a rigorous evaluation of DGMB-Net’s performance and benchmarked it against several baseline models. The experimental outcomes provided unequivocal validation of the effectiveness and robustness of DGMB-Net.

Bayonet-Drivers provides a new benchmark and suitable data for the development of the transport industry. Simultaneously, DGMB-Net can effectively reduce the workload of human monitoring and labeling for traffic management departments by means of semi-supervision. Looking forward, on the one hand, a lightweight network design will be incorporated to enhance DGMB-Net’s efficiency. On the other hand, additional types of risky driving behaviors, such as drowsy driving, drunk driving, smoking, and eating, among others, will be added to broaden the scope of the research. Additionally, the application of unsupervised learning will be applied in the identification of risky behaviors. This expanded focus will undoubtedly bolster the applicability of the detection network within Intelligent Transportation Systems.

## Figures and Tables

**Table 2:** Detailed information about Bayonet-Drivers.

Using Mobile Phones	Seat Belt Violation	Safe/Unsafe	Number of Images
No	Yes	Unsafe	2500
Yes	No	Unsafe	2500
Yes	Yes	Unsafe	2500
No	No	Safe	2500

**Table 3:** Ablation experiment results of APL module on Bayonet-Drivers dataset. The best result are highlighted in bold.

Dataset	Backbone	+DCN	+GC	mAP	mAP@0.5	mAP@0.75	$AP_{M}$	$AP_{L}$
Bayonet-Drivers	ResNet18			49.4	84.9	53.4	48.1	47.8
		✓		50.1	85.6	54.4	48.7	50.1
			✓	50.3	85.7	55.6	48.9	53.6
		✓	✓	51.5 (+1.1)	86.2 (+1.3)	56.3 (+2.9)	49.3 (+1.2)	56.3 (+8.5)
	ResNet50			50.1	84.3	55.4	48.3	55.5
		✓		50.4	86.5	56.1	48.8	55.7
			✓	51.5	86.9	57.6	49.1	61.3
		✓	✓	**53.5 (+3.4)**	87.1 (+2.8)	**57.8 (+2.4)**	50.1 (+1.8)	**62.4 (+6.8)**
	ResNet101			49.8	84.7	55.3	49.1	55.3
		✓		50.1	85.6	54.4	48.7	50.1
			✓	51.3	87.1	57.4	50.1	60.3
		✓	✓	52.9 (+3.1)	87.5 (+2.8)	57.5 (+2.1)	**50.5 (+1.4)**	61.4 (+6.1)
	ResNeXt101			50.1	84.8	55.3	49.3	55.4
		✓		50.5	87.0	56.0	49.5	55.8
			✓	51.3	87.2	57.5	49.9	60.4
		✓	✓	53.5 (+3.4)	**87.6 (+2.8)**	57.7 (+2.4)	50.4 (+1.1)	62.2 (+6.8)

**Table 4:** Ablation experiment results of APL Module on State Farm dataset. The best result are highlighted in bold.

Dataset	Backbone	+DCN	+GC	mAP	mAP@0.5	mAP@0.75	$AP_{M}$	$AP_{L}$
State Farm	ResNet18			67.6	99.0	84.2	73.4	70.6
		✓		68.4	99.3	85.6	74.6	71.9
			✓	68.6	99.4	84.9	74.8	72.2
		✓	✓	69.4 (+1.8)	99.5 (+0.5)	86.2 (+2.0)	74.9 (+1.5)	72.5 (+1.9)
	ResNet50			69.5	99.4	87.9	64.5	76.0
		✓		71.0	99.7	88.1	67.7	76.4
			✓	71.3	99.7	87.5	67.4	77.2
		✓	✓	**71.3 (+1.8)**	99.8 (+0.4)	**91.1 (+3.2)**	67.8 (+3.3)	**77.8 (+1.8)**
	ResNet101			67.5	98.8	83.9	75.8	67.7
		✓		68.7	99.2	85.6	76.9	69.8
			✓	69.4	99.5	86.3	77.1	68.7
		✓	✓	70.8 (+3.3)	**99.9 (+1.1)**	87.0 (+4.9)	77.8 (+2.0)	70.8 (+3.1)
	ResNeXt101			68.7	99.1	85.3	76.5	72.0
		✓		69.8	99.3	85.8	77.1	72.5
			✓	70.1	99.5	86.2	77.3	72.8
		✓	✓	71.2 (+2.3)	**99.9 (+0.8)**	90.2 (+4.9)	**77.9 (+1.4)**	74.5 (+2.5)

**Table 5:** Performance comparison between HFPN and different FPNs on Bayonet-Drivers dataset. The best result are highlighted in bold.

Dataset	Backbone	FPN	*AP*	*mAP*@0.5	*mAP*@0.75	$AP_{M}$	$AP_{L}$	Params (M)
Bayonet-Drivers	ResNet50	-	50.1	84.3	55.4	48.3	55.6	372.52
	ResNet50	FPN [58]	51.4	85.6	56.0	49.5	54.0	68.93
	ResNet50	BiFPN [59]	52.3	87.5	58.9	50.6	44.1	70.11
	ResNet50	PAFPN [54]	51.6	86.1	57.2	49.5	55.0	72.47
	ResNet50	BFP [55]	51.9	86.7	57.7	49.5	47.0	69.19
	ResNet50	HFPN	**53.4 (+3.3)**	**87.8 (+3.5)**	**58.9 (+3.5)**	**50.8 (+2.5)**	**56.5 (+0.9)**	72.73

**Table 6:** Performance comparison between HFPN and different FPNs on State Farm dataset. The best result are highlighted in bold.

Dataset	Backbone	FPN	*AP*	*mAP*@0.5	*mAP*@0.75	$AP_{M}$	$AP_{L}$	Params (M)
State Farm	ResNet50	-	69.5	99.4	87.9	64.5	76.0	372.52
	ResNet50	FPN [58]	70.6	99.6	88.2	65.4	76.2	68.93
	ResNet50	BiFPN [59]	70.8	99.5	88.1	64.8	76.1	70.11
	ResNet50	PAFPN [54]	70.8	99.6	88.3	65.6	**76.9**	72.47
	ResNet50	BFP [55]	70.9	99.7	89.5	64.9	76.7	69.19
	ResNet50	HFPN	**71.2 (+1.7)**	**99.8 (+0.4)**	**89.9 (+0.4)**	**65.7 (+1.2)**	**76.9 (+0.9)**	72.73

**Table 7:** Comparative experimental results of DGMB-Net with other semi-supervised methods on Bayonet-Drivers dataset and State Farm dataset. The best result are highlighted in bold.

Dataset	Method	1%	5%	10%	20%	30%	GFlops
Bayonet-Drivers	supervised	10.3	14.6	39.2	47.1	49.3	-
	CSD [60]	13.5 (+3.2)	15.4 (+0.8)	39.4 (+0.2)	47.2 (+0.1)	49.2 (−0.1)	234.47
	STAC [51]	22.6 (+12.3)	28.2 (+13.6)	46.4 (+7.2)	50.7 (+3.6)	49.6 (+2.1)	234.47
	Humble Teacher [61]	29.6 (+19.3)	33.8 (+19.2)	47.8 (+8.6)	51.3 (+4.2)	49.9 (+3.3)	234.47
	E2E [62]	33.5 (+23.2)	37.5 (+23.0)	49.9 (+10.7)	52.6 (+5.5)	53.3 (+3.0)	234.47
	DGMB-Net	**36.0 (+26.7)**	**39.8 (+25.2)**	**51.0 (+11.8)**	**53.8 (+6.7)**	**54.5 (+5.2)**	234.47
State Farm	supervised	2.7	20.6	60.5	62.6	68.4	-
	CSD [60]	6.2 (+3.5)	22.8 (+2.2)	60.8 (+0.3)	62.8 (+0.2)	68.5 (+0.1)	234.47
	STAC [51]	10.6 (+7.9)	25.6 (+5.0)	63.9 (+3.6)	65.1 (+2.5)	69.6 (+1.2)	234.47
	Humble Teacher [61]	12.8 (+10.1)	26.9 (+6.3)	65.3 (+4.8)	65.8 (+3.2)	70.1 (+1.7)	234.47
	E2E [62]	14.9 (+12.2)	28.8 (+8.2)	66.6 (+6.1)	66.9 (+4.3)	71.2 (+2.8)	234.47
	DGMB-Net	**16.6 (+13.9)**	**30.7 (+10.1)**	**68.7 (+8.2)**	**69.4 (+6.8)**	**72.3 (+3.9)**	234.47

**Table 8:** Comparison results of DGMB-Net and mainstream object detection algorithms on Bayonet-Drivers dataset. The best result are highlighted in bold.

Dataset	Model	Backbone	*AP*	*mAP*@0.5	*mAP*@0.75	$AP_{M}$	$AP_{L}$	Params (M)
Bayonet-Drivers	Fast RCNN [65]	ResNet50	36.3	78.9	31.2	39.6	25.6	40.53
	Faster RCNN [66]	ResNet50	46.9	83.9	48	44.7	48.4	41.13
	Yolox [63]	DarkNet	41.4	82.9	33.2	41.0	22.4	54.21
	Retinanet [64]	ResNet50	50.2	86.3	52.9	48.1	76.0	37.74
	Cascade RCNN [67]	ResNet50	48.5	83.3	51.6	46.3	29.2	68.93
	RDB-Net	ResNet50	51.2	87.1	53.4	48.6	77.1	74.82
	DGMB-Net	ResNet50	**54.5**	**88.3**	**54.6**	**49.3**	**78.2**	74.82

**Table 9:** Comparison results of DGMB-Net and mainstream object detection algorithms on State Farm dataset. The best result are highlighted in bold.

Dataset	Model	Backbone	*AP*	*mAP*@0.5	*mAP*@0.75	$AP_{M}$	$AP_{L}$	Params (M)
State farm	Fast RCNN [65]	ResNet50	48.6	84.3	43.8	45.3	49.4	40.53
	Faster RCNN [66]	ResNet50	65.9	98.0	87.5	65.2	75.6	41.13
	Yolox [63]	DarkNet	64.9	97.9	79.2	59.5	70.1	54.21
	Retinanet [64]	ResNet50	65.4	99.1	82.1	60.6	70.2	37.74
	Cascade RCNN [67]	ResNet50	66.8	99.0	90.1	68.5	77.8	68.93
	RDB-Net	ResNet50	68.6	99.3	90.5	69.6	78.8	74.82
	DGMB-Net	ResNet50	**72.3**	**99.9**	**91.3**	**70.2**	**79.6**	74.82

## Data Availability

You can access the dataset from this research at the link https://github.com/HuanYingLi/Dataset-for-Risky-Driving-Detection (accessed on 25 November 2023).

## Abbreviations

The following abbreviations are used in this manuscript:

ITS	Intelligent Transportation Systems
APL	Adaptive Perceptual Learning
HFPN	Hierarchical Feature Pyramid Network
WHO	World Health Organization
LBP	Local Binary Pattern
HOG	Histogram of Oriented Gradients
SVM	Support Vector Machines
CNN	convolutional neural network
FPN	feature pyramid network
NMS	Non-Maximum Suppression
NWPG	Nonlinear Weighted Pseudo Boxes Generation
GC	global context
DCN	deformable convolution
ECA	Efficient Channel Attention
RPN	Region Proposal Network
IoU	Intersection over Union
PR Curve	precision–recall curve
ROC	Receiver Operating Characteristic
